# Evaluation of the impact of interdisciplinarity in cancer care

**DOI:** 10.1186/1472-6963-11-144

**Published:** 2011-06-03

**Authors:** Dominique Tremblay, Danièle Roberge, Linda Cazale, Nassera Touati, Elizabeth Maunsell, Jean Latreille, Jacques Lemaire

**Affiliations:** 1 Charles LeMoyne Hospital Research Center, Greenfield Park, Québec, Canada; 2 School of Nursing, Université de Sherbrooke (Longueuil campus), Longueuil, Québec, Canada; 3 Department of Community Health Sciences, Université de Sherbrooke (Longueuil campus), Longueuil, Québec, Canada; 4 Department of Health Statistics, Institut de la statistique du Québec, Montréal, Québec, Canada; 5 École nationale d'administration publique, Montréal, Québec, Canada; 6 Faculty of Medicine, Université Laval, Québec City, Québec, Canada; 7 Faculty of Medicine and Health Sciences, Université de Sherbrooke (Longueuil campus), Longueuil, Québec, Canada; 8 Centre intégré de cancérologie de la Montérégie, Greenfield Park, Québec, Canada

## Abstract

**Background:**

Teamwork is a key component of the health care renewal strategy emphasized in Quebec, elsewhere in Canada and in other countries to enhance the quality of oncology services. While this innovation would appear beneficial in theory, empirical evidences of its impact are limited. Current efforts in Quebec to encourage the development of local interdisciplinary teams in all hospitals offer a unique opportunity to assess the anticipated benefits. These teams working in hospital outpatient clinics are responsible for treatment, follow-up and patient support. The study objective is to assess the impact of interdisciplinarity on cancer patients and health professionals.

**Methods/Design:**

This is a quasi-experimental study with three comparison groups distinguished by intensity of interdisciplinarity: strong, moderate and weak. The study will use a random sample of 12 local teams in Quebec, stratified by intensity of interdisciplinarity. The instrument to measure the intensity of the interdisciplinarity, developed in collaboration with experts, encompasses five dimensions referring to aspects of team structure and process. Self-administered questionnaires will be used to measure the impact of interdisciplinarity on patients (health care utilization, continuity of care and cancer services responsiveness) and on professionals (professional well-being, assessment of teamwork and perception of teamwork climate). Approximately 100 health professionals working on the selected teams and 2000 patients will be recruited. Statistical analyses will include descriptive statistics and comparative analysis of the impact observed according to the strata of interdisciplinarity. Fixed and random multivariate statistical models (multilevel analyses) will also be used.

**Discussion:**

This study will pinpoint to what extent interdisciplinarity is linked to quality of care and meets the complex and varied needs of cancer patients. It will ascertain to what extent interdisciplinary teamwork facilitated the work of professionals. Such findings are important given the growing prevalence of cancer and the importance of attracting and retaining health professionals to work with cancer patients.

## Background

### Objective, Context and Research Questions

The general objective of this project is to evaluate the effects of interdisciplinarity (hereafter referred to as ID) on both cancer patients and health professionals. Interdisciplinary teamwork is a health care renewal strategy promoted in a number of forums for enhancing the quality of both primary and specialized care [1-6]. Most cancer programs, not only in Quebec [7-12] but also in Canada [13-16] and around the world, [17-23] are introducing ID as a key modality. Although the literature suggests some positive outcomes of ID, it does not identify how its intensity and its variation affect outcomes [3,6], and knowledge is rather limited on the benefits of the interdisciplinary cancer team model advocated in many Canadian provinces.

The interdisciplinary teamwork model implemented in Quebec's local cancer teams builds on the synergy of professionals with different disciplinary backgrounds who work together to provide medical treatments and supportive patient-centred care. These teams are located in hospital ambulatory clinics and collaborate with providers from other settings. To update the cancer program in Quebec, considerable efforts are being made to introduce and consolidate ID and to promote a formal team accreditation process [12]. The accreditation reviews of 65 local teams have been completed. According to Ministry officials responsible for the on-site accreditation process, ID varies widely in terms of both team composition and operating methods, depending notably on the care environment. Teams performing real-life tasks in natural settings offer a unique opportunity to better understand the critical factors of ID associated with team outputs [24,25].

Our research project will be guided by the following questions: a) What are the effects of ID intensity on three specific patient outcomes: use of cancer-related services, perception of the degree to which service needs were met and perception of care and service quality? b) What are the effects of ID intensity on three outcomes among health professionals: perceived team effectiveness, job satisfaction and perceived well-being? c) To what extent are outcomes influenced by characteristics of patients, professionals and the work environment? d) What are the critical factors (work environment, team structure, team process) that contribute to ID intensity (weak, moderate, or high)?

The combined knowledge stemming from this research and from ongoing Ministry of Health monitoring will support more thorough assessment of ID as a key component of cancer services reform, both for the scientific community and for decision-makers throughout the health care system. Our project is in line with previous research conducted by members of our team on the reorganization of cancer services [26,27].

### Interdisciplinary teamwork definition and intensity classification

Interdisciplinary teamwork is defined as work done by a group of people with various expertises, responsible for individual decisions, who hold a common purpose and meet together to communicate, share and consolidate knowledge from which plans are made [28]. Team composition varies in number and diversity of disciplines. Some research suggests that as ID intensifies, so does the impact on patients, health professionals and organizations [2,29,30]. However, while the classification and selection of teams according to ID intensity are crucial factors in the validity of such studies, few tools finely evaluate teamwork intensity [2,31]. Research by West et al. [32] seems especially useful for such evaluation and figures prominently in summaries of conceptual work on health care teamwork [31-34]. According to West's seminal work, structural and process-related aspects must be considered when assessing ID. Structural characteristics refer primarily to team composition and size. Process-related factors describe interactions among team members, in particular a shared philosophy of care, leadership, coordination within the team and with partners, and the team's orientation in terms of quality evaluation [29,32,34-36].

### Factors that can affect interdisciplinarity and team impact

Three broad groups of factors can simultaneously or separately affect interdisciplinarity and team impact. The first is *characteristics of the work environment*. These include organizational and managerial attributes, i.e., the degree of specialization of the hospital/center where the team is located, its geographic location (rural or urban), an organizational culture favourable to ID, institutional support for work environments (premises, information technologies), and clear leadership with respect to the implementation of ID [2,35,37-39]. Workload must also be considered, since it can affect resource availability, services offered and patient satisfaction [29,40]. The nature of collaboration between the teams and community facilities can also affect teams' ability to make referrals and patients' timely access to such facilities. However, relationships among these variables are complex and study findings are sometimes contradictory. The second group of factors is *patient characteristics*. Age, level of education, socio-economic standing and perceived state of health are the most frequently considered variables [41-43]. These are important when measuring perception of care and when evaluating whether interdisciplinary intensity has a main effect on patient outcomes. Perceived quality of life and clinical issues such as stage in the cancer trajectory and type of treatment should also be taken into account. Finally, the third group is *professional characteristics*, i.e., the disciplines represented, each professional's training and work experience in ID, and responsibility for the team's development. All these characteristics appear to affect professionals' perceptions of their team's effectiveness as well as their job satisfaction [2,29,44,45].

### Outcomes of interdisciplinary teamwork

There is evidence supporting benefits of ID for patients/clients, providers and the system overall in specialized areas such as chronic disease prevention and management [3,6]. Most studies have been descriptive and have tended to focus on team characteristics (*who*) and processes (*what they do and how) *rather than on outcomes for either team members or patients/clients [2,3,5,31,46-49]. Moreover, the studies often display significant limitations with respect to the concept of ID [2,48-50]. Some focus on the doctor-nurse relationship [51] or only on teamwork among different medical specialists (oncologist, surgeon, pathologist) [52]. Some studies have focused on the outcomes of interdisciplinary teams providing primary care [53-56] or working in mental health or with elderly people experiencing a loss of autonomy [5,39,40]. Literature reviews on teamwork involving different clienteles have also appeared recently [2,3,6,47-49]. This research reveals that patient benefits include enhanced management of symptoms, broader access to services, improved functional status and increased satisfaction with care. The impacts reported for professionals are positive and negative. While enhanced job satisfaction, improved mental health and increased efficiency in the work accomplished have all been noted, so also have more extensive interpersonal conflicts. In organizations, the impacts observed include more efficient use of resources, e.g. reduced reliance on emergency services, less absence from work, and broader access to care. A bilateral approach that includes assessment of both patients and health professionals is recommended for a more comprehensive assessment of teamwork impact [45,47,57].

In oncology, the impact of cancer therapy committees and palliative care teams on quality of care has been assessed [29,58-64]. However, those findings are hardly applicable, as the type of teams studied are different from the one that is the subject of this proposal in terms of focus (therapy vs. supportive care) and stage in the cancer trajectory (acute vs. palliative). Studies on interdisciplinary cancer teamwork mainly report on implementation [65], interdisciplinary functioning and decision-making [66,67] of teams of medical specialists or of doctor/nurse collaborations [30,52].

### Research hypotheses

The study seeks to test two main hypothesis: (1) the greater the intensity of ID, the more extensive are the beneficial effects experienced by patients and health professionals; and (2) the potential benefits of teamwork differ depending on the characteristics of the patients being treated, the professionals, and the care environments.

### Conceptual framework

**Figure 1:**
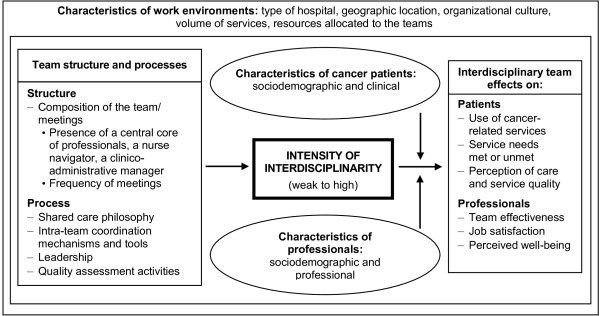
**Conceptual framework relating interdisciplinary teamwork to patient and professional outcomes**.

Figure 1 illustrates the conceptual framework of the study, which builds upon commonly accepted frameworks on team effectiveness, including the work of West et al. [24,32,46,68] The convergence of team structure and process components results in the intensity of ID. The intensity of ID is associated with potential outcomes for patients (use of health services, service needs met or unmet, perception of quality of care and services) and for professionals (team effectiveness, job satisfaction and perceived well-being). The framework reveals that characteristics of the care environments of local teams can affect the intensity of ID and the potential outcomes. Similarly, the characteristics of cancer patients and health professionals can mediate the outcomes observed.

### Description of the intervention: local interdisciplinary cancer teams

The notion of ID advocated in Quebec by the Ministry's consultative committee [7] resembles that promoted in Canada and elsewhere in the world. According to that committee, ID involves grouping together several different health care professionals, each with specific training and skills, who work together to achieve concerted intervention and the complementary sharing of tasks. Three types of interdisciplinary teams are planned in Quebec [8,10,11], i.e., local, regional and supraregional--the two latter being distinguished by the degree of specialization of the services offered and their consulting role. Our study focuses on local teams being established in all hospitals offering oncology services (n = 65). Most of the local teams provide supportive care and treatments to patients who come to the outpatient clinics for investigative examinations, chemotherapy, and follow-up visits. These teams differ from cancer therapy committees and specialized palliative care teams. Cancer therapy committees, made up primarily of medical staff, are discussion forums that examine diagnostic and therapeutic options for complex clinical cases [69]. Palliative care teams usually intervene in specialized areas for patients who need end-of-life home or institutionalized care [70].

Local teams should include a core of professionals, including a pivot nurse (also called nurse navigator), a pharmacist, a medical oncologist, a nutritionist, and a social worker or a psychologist [7]. The number and diversity of professionals remains to be specified in relation to the needs and volume of clients. Interdisciplinarity involves: a comprehensive initial assessment of patients' needs from a holistic perspective; formal and regular interdisciplinary meetings to discuss the cases of patients (and relatives) experiencing complex biopsychosocial situations; the development of concerted interdisciplinary intervention plans; the mastery of coordination procedures and tools, both within the teams and with partners upstream and downstream from oncology outpatient clinics; and the implementation of measures to ascertain the quality of the services offered.

### Pilot study

This research project is based on a pilot study. A detailed report (in French) of that study is available on the Internet [71]. Briefly, the pilot study first sought to develop a tool to measure the intensity of interdisciplinarity. To develop that tool, we consulted four professionals, four managers and a researcher, all recognized for their expertise in the content and practice of interdisciplinary teamwork and in cancer care. They were asked to rate the importance in oncology of the components of ID that were most often reported in our extensive literature review. These experts deemed the following aspects to be important: (1) team composition and frequency of meetings; (2) clinico-administrative responsibility; (3) a shared philosophy of care; (4) coordination mechanisms and tools; and (5) quality of care evaluation activities. Based on the experts' opinions, each facet was given equal weight and the total score of ID intensity ranges from 0 to 10.

The pilot study also sought to develop and pre-test two tools to measure the potential impacts of ID, one for patients and one for health professionals. They were designed to integrate the measurement scales with demonstrated psychometric qualities. New questions were developed and added in order to cover all of the potential impacts of ID on patients and professionals. The validity of each questionnaire's content (clarity of items, completeness) was pre-tested with 10 cancer patients visiting an oncology clinic in a regional hospital in the province of Quebec, and with 10 health professionals from two local teams in the same region. Respondents' comments were incorporated into the final versions of the questionnaires. Patients took 35 minutes, on average, to fill out the questionnaire and professionals, 30 minutes [71].

## Methods/Design

### Research design

An experimental design is impossible because every cancer team in Quebec was encouraged to develop ID during the first round of accreditation visits in 2007. Consequently, this study will take advantage of natural variations in the intensity of implemented ID in local teams to measure the effects on outcomes. The proposed design therefore consists of an ex post quasi-experimental study type [72], which will compare three groups that differ with respect to their intensity of ID: (1) weak; (2) moderate; and (3) high.

#### Target and study populations

The target population comprises the 65 oncology outpatient clinic care teams (local teams) in Quebec. The study population will comprise a total of 12 local teams randomly selected within strata established on the basis of ID intensity, i.e., high, moderate or weak. Four local teams will be selected in each stratum. Based on our experience with the pilot study and our previous organizational research conducted in hospitals, we expect most will agree to participate in the study.

#### Definition of the independent variable and operationalization

**Figure 2:**
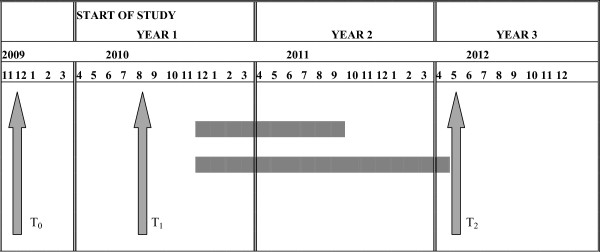
**Measurements of the level of interdisciplinarity of teams**. T_0 _: Classification of all local teams in the province (n = 65) in relation to their level of interdisciplinarity T_1 _: Validation of the level of interdisciplinary intensity of the selected teams (n = 12) T_2 _: Update of the level of interdisciplinary intensity of the participating teams (n = 12)

The independent variable is the intensity of interdisciplinary teamwork. It includes five aspects of team structure and processes. The score for the interdisciplinary teamwork construct ranges theoretically from 0 to 10. The pilot study suggests that the lowest-scoring teams are those that appear to be the least structured and whose work processes involve minimal collaboration. In contrast, teams with high ID have structures more closely aligned with the preferred model in terms of professional diversity and regularity of meetings, and their processes are shared and diversified. Figure 2 illustrates the points at which the local teams' levels of ID will be documented. First (T_0_), the levels will be measured for all local teams in Quebec (n = 65) using the Ministry of Health's database, which includes information collected in all Quebec hospitals in 2006-2007 during the first round of accreditation visits to local cancer teams.

We will assess the concurrent validity of our ID measurement tool using a subscale of interdisciplinary collaboration applicable to primary care services [38]. This subscale consists of seven items that measure interdisciplinary coordination (Cronbach's α coefficient: 0.82). The distribution of the team ratings will be divided into tertiles in ascending order of intensity (weak, moderate, high). Then we will randomly oversample five teams in each stratum to ensure, after validation, a sample of four teams per stratum (n = 12). Their level of ID will be validated by an update at the start of the study (T_1_). A second update will be done 18 months later, at the end of the patients' and professionals' data collections (T_2_). The teams that will be included in the study at T_1 _are those whose rating is relatively stable (± 10%) between T_1 _and T_0_. Changes in ID levels between T_1 _and T_2 _will be taken into account through sensitivity analyses on dependent variables.

#### Definition of dependent variables and operationalization

The dependent variables are the outcomes for patients and professionals. We anticipate impacts on patient outcomes will be seen in the use of cancer-related services, the perceived degree to which service needs were met and the perceived quality of care and services. These constructs will be measured by means of a self-administered questionnaire using closed-ended questions. English and French versions of the questionnaires will be available. Translation processes in English or French will follow the seven-step system developed by Vallerand [73].

Our patient questionnaire comprises four sections. The **first section **focuses on service use over the past year. It includes 22 questions to document the services used by the patient (e.g. unscheduled visits to family doctor or emergency) and all their service needs, whether met or not by the oncology clinic. It is partly based on a recent questionnaire elaborated by Pineault et al. [74] to document the public's experience of primary care use. The **second section **documents the patient's perceptions of the quality of care and services. It includes 34 items divided into six subscales. Three of these subscales come from the WHO's Health Systems Responsiveness Questionnaire [75]. This instrument has been translated, adapted to cancer services and validated by a member of the research team [26]. The reliability of these subscales (Cronbach's α) ranges from 0.69 to 0.83. The three other subscales (accessibility, continuity of care, care outcomes) are based on the Picker questionnaire for cancer care [76] and that of Pineault et al. [74]. The factorial analysis and reliability of these subscales will be evaluated in the study. The **third section **includes a measure of the patient's quality of life. The French version of the fourth edition of the *Functional Assessment of Cancer Therapy *(FACT-G) will be used [77-80]. The FACT-G comprises 27 items that measure physical, social and familial, emotional and functional well-being. The reliability of these subscales (Cronbach's α) ranges from 0.77 to 0.90. The **fourth section **of the questionnaire includes 26 questions that document respondents' sociodemographic and clinical characteristics.

In terms of outcomes for health professionals, the potential impacts relate to perceived team effectiveness, job satisfaction and well-being. These impacts will likewise be measured by means of a self-administered questionnaire using closed-ended questions.

The questionnaire for professionals comprises three sections. The **first section **contains 26 items and focuses on an assessment of teamwork and job satisfaction. It includes four validated, reliable subscales (Cronbach's α coefficient: 0.82 to 0.91). The first subscale focuses on the professional's perception of the team's functioning [81]. The second subscale measures the professional's perception of the team's cohesiveness [5]. The other two subscales measure the professional's satisfaction with teamwork [39] and perception of the team's results [38]. The **second section **of the questionnaire deals with the professional's perceived well-being. We will use the French version of the Maslach Burnout Inventory (MBI) [82,83]. This scale, made up of 22 items, is the most widely used standardized measure of professional burnout [84] and was recently used in conjunction with Canadian surveys of health professionals working in cancer care [61,84]. This questionnaire comprises three facets: emotional burnout (α = 0.90), depersonalization (α = 0.79) and professional achievement (α = 0.72). The **third (last) section**, comprising 12 questions, documents the respondent's sociodemographic and professional characteristics.

#### Secondary independent variables

Several characteristics of patients, professionals and the care environment are likely to affect patients' and professionals' perceptions of the care experience, whether independently or through a confounding effect on the effects of interdisciplinarity. These variables are: (1) **cancer patient characteristics **such as age, level of education, sex, economic status, quality of life, tumour site and stage (metastatic, non-metastatic), co-morbidity, perceived state of health at the time of diagnosis and at the present time, treatment received, prior care experience, presence of a family member or other caregiver, and case management by the pivot nurse; (2) **health professional characteristics **such as age, sex, level of education, professional experience, number of years on the team, time devoted to the team, and professional category; and (3) **work environment characteristics **such as the hospital's degree of specialization (research, teaching, radiotherapy services), geographic location (urban or rural), volume of services offered in oncology outpatient clinics, resources allocated to the teams (e.g. training, information systems), support for the teams, and collaboration between the team and local community health organizations.

#### Data collection

To ascertain the intensity of ID at T_0_, T_1 _and T_2 _(Figure 2), we will use the assessment grid developed in the pilot project. At each measurement point, data will be collected from the local team managers by means of postal surveys. The grid will be mailed to them accompanied by a cover letter, a consent form and a stamped reply envelope. Two reminders will be sent at one-month intervals [85]. A second set of data will be collected through telephone interviews of team managers to document the characteristics of care environments. Variables describing care environments will be documented from hospital administrative data. Based on our experience during the pilot study, the refusal rate should be minimal or nil.

##### Survey of cancer patients

Patients will be recruited during visits to oncology outpatient clinics. The inclusion criteria of this convenience sample will be kept to a minimum to ensure the subjects' broad representativeness. To be included in the sample, subjects must be 18 years of age or over, have visited an oncology outpatient clinic at least once in the preceding 12 months, and read and understand French or English. Designated clerks will distribute the questionnaires; they will first be given relevant information on the project and receive written instructions to ensure the questionnaires are presented in a uniform manner. The patients will be asked to fill out the questionnaire at home and return it by mail in the stamped reply envelope. To encourage the distribution and submission of the questionnaires, the clerks will receive follow-up calls, and posters bearing reminders for patients will be displayed in waiting rooms. Reminders will also be sent to the patients' homes at two and four weeks post-distribution. According to Dillman [85] and studies carried out by members of our team using the same proactive strategy, a response rate of 70% can be expected [26,86].

##### Survey of professionals

Managers will be asked to provide a list of all the professionals on the cancer team and to invite them to participate in the project. Then the study coordinator will contact team members to obtain their participation. Haward et al. [29] successfully adopted a similar approach. Respondents to the questionnaire must have a minimum of three months of experience in the oncology outpatient clinic [81]. A representative of the research team will distribute the questionnaires and related documents (cover letter and consent form) at a meeting with the team. The managers will not know who is or is not participating. Two reminders will be sent to non-respondents at one-month intervals. With this approach, a response rate of 90% is expected. Based on our previous studies, the rate of participation among physicians is expected to be lower than for other professionals.

### Analysis

The impacts of ID intensity on the patient's outcomes (research question (a)) and health professional's outcomes (research question (b)) will be analysed *separately *according to the hierarchical structure (sites/patients or sites/professionals) of our data using multilevel analyses [87]. Most of these dependent variables, such as "use of cancer-related services" for the patients and "job satisfaction" for the professionals, have quantitative scales adequate for means comparison between levels of intensity. Normality and homogeneity of variance tests will be carried out. Therefore, we will use mainly two-level ANOVA models to test *separately *whether patients' or health professionals' quantitative outcomes differ among the levels (groups) of intensity. Some of the outcomes may show skewed or inadequate distributions for ANOVA analyses. For these, (1) adequate mathematical transformations will be tried to achieve normality and homogeneity, or (2) for some quantitative discrete outcomes, such as "use of cancer-related services (e.g. number of visits at emergency)", Poisson regression will be used with dummy variables (interdisciplinarity). If we fail to achieve (1) or execute (2), those variables will be recoded in a dichotomous variable (e.g. "low" versus "high" according to median). For any naturally dichotomous outcomes, such as "unsatisfied service needs" for the patient, or the recoded variables, multilevel logistic regression will be conducted. ANOVA analyses, Poisson and logistic regressions can account for confounding effects or main effects of secondary variables, whether quantitative (e.g. "patient's age or level of education") or qualitative (e.g. "the hospital's degree of specialization"), as planned in research question (c). Finally, to address research question (d), we will use structural equation modelling [88,89] to validate and assess the matching of the conceptual framework presented in Figure 1 to our observations. Before inferential analyses, we will undertake a series of descriptive statistical analyses of ID components of the conceptual framework (Figure 1) and the attendant characteristics (means, standard deviations, cross-tabulations, etc.) and item-by-item analyses of patients' perceptions of the quality of care.

Furthermore, we will assess the psychometric qualities of the subscales developed for this study by means of fidelity analyses (internal consistency) and validity analyses, e.g. exploratory factor analysis [90].

#### Sample size

At the site level, feasibility constraints prevent us from studying more than 12 teams (3 levels of intensity * 4 teams). For this reason, power for detecting differences according to level of ID intensity using oncology outpatient clinic care teams as the analytic unit cannot be the primary concern; our approach will be exploratory. Since all the professionals must participate (anticipated rate of participation is 90%), we will randomly oversample five teams in each level of intensity to ensure, after validation, the expected sample size of 12 teams. For the nested professional level, we have to deal with the limited number of professionals in a team. Based on a minimum of eight participating professionals per team, we will likely have more than 96 (3 levels * 4 teams * 8) professionals in our sample. For the nested patient level, we will oversample to compensate for the response rate. Main analyses consist of ANOVA and logistic regression. Power calculations for single-factor (three-level) simple modeling (not mixed-model) ANOVA analyses reveal that to detect a relative effect size of 0.25 and a power of 90%, a total sample of 1218 patients is indicated. The possible presence of an intraclass correlation imposes the correction of these sizes. If we consider an analysis of dichotomized scores using simple modeling logistic regression, based on the conservative scenario in which power = 80%, reference proportion = 15%, and odds ratio (OR) = 1.5, we would need a total sample size of 1968. This final sample of 1968 (3 levels * 4 teams * 164) patients allows us to take into account structural equation models with 100-odd parameters [91,92].

## Ethical considerations

The protocol has been approved by the ethics boards of Charles LeMoyne Hospital and all other participating hospitals (MP-HCLM-09-050).

## Discussion

### Internal and external validity of the study

We have taken several precautions to ensure the study's internal validity. To minimize the occurrence of a maturation bias of the principal independent variable, we will measure the level of ID when teams are selected (T_0_), and update it at the beginning (T_1_) and when the patient and health professional data collections are completed (T_2_). Moreover, we have taken steps to minimize the occurrence of a selection bias or of bias through distorting effects stemming from differences between non-equivalent comparison groups. We have made provision to control for the confounding effects of an array of variables related to patients, professionals and care environments by means of detailed data collection and reliance on appropriate statistical methods. Based on the pilot study and on previous research using the same approach, we anticipate that the refusal rate among the local teams invited to participate will be minimal. Our data collection relies on tools that have been validated and extensively used in research, and we have adopted strategies to optimize the response rate. While an experimental design would have been optimal to evaluate the research question, it cannot be envisaged in the current cancer services, as the majority of local programs promote and support interdisciplinary team work. However, the scarcity of studies published to date on the effect of variation of interdisciplinary teams in oncology justifies our proposed approach.

The possibility of extending findings to other populations or settings can be jeopardized when the causal relationship is ambiguous. In this study, the robustness of the links between the independent and dependent variables is reinforced by the proposed conceptual framework. External validity can also be jeopardized by interaction between the intervention and the experimental situation. For this study, we will have access to the entire array of local descriptive data recorded in Ministry of Health accreditation records, as well as the collaboration of departmental officials responsible for monitoring the implementation of the teams. In this way, we will gain a broader understanding of the conditions under which the results can be extended to other local team models or settings.

#### Relevance of the research project and expected results

This research will provide evidence to foster reflection on ID as a means of organizing cancer services. More specifically, the study will enable us to pinpoint to what extent interdisciplinary teamwork enhances quality of care and meets the complex and varied needs of cancer patients. It will provide relevant information on the impact of ID on health professionals working with cancer patients. These findings are especially useful in a context where the attraction and retention of professionals is a key concern. The study will provide information on the conditions under which the results can be extended to other populations and settings, which is particularly useful to professionals in the health care system interested in adopting ID. From a scientific standpoint, this research will enrich both the notion of the intensity of ID and knowledge on the benefits of this approach to intervention in the realm of cancer.

#### Dissemination of the findings

Our dissemination strategies are based on evidence produced by experts in knowledge translation [93-96]. We will emphasize ongoing interactions between researchers, policy-makers, managers and clinicians. We will pursue the exchanges initiated during the pilot study and the development of this research proposal by establishing a project advisory committee made up of representatives of decision-making and professional bodies involved in implementing interdisciplinary teams as mandated by the cancer program. The committee's composition will be determined with project collaborators. The committee will act as a forum for discussions on the project's orientation, its findings and conclusions, and any spin-offs with respect to dissemination. We also envisage conventional means of dissemination, such as distribution of the research report among the bodies concerned, presentations at scientific conferences, and publication of articles in recognized scientific journals. Finally, our knowledge transfer and exchange activities will target decision-makers and health professionals. We will publish results in management and professional journals, and will rely on our knowledge exchange relationships for communicating results to decision-makers through regional and Canadian professional conferences.

## Competing interests

The authors declare that they have no competing interests.

## Authors' contributions

DT and DR were responsible for drafting the manuscript. All authors reviewed and accepted the final version of the manuscript.

## Pre-publication history

The pre-publication history for this paper can be accessed here:

http://www.biomedcentral.com/1472-6963/11/144/prepub
